# The influence of warming on the biogeographic and phylogenetic dependence of herbivore–plant interactions

**DOI:** 10.1002/ece3.4918

**Published:** 2019-01-28

**Authors:** Xidong Mu, Meng Xu, Anthony Ricciardi, Jaimie T. A. Dick, Du Luo, Hui Wei, Yinchang Hu, Qiwei Wei

**Affiliations:** ^1^ College of Fisheries Huazhong Agricultural University Wuhan China; ^2^ Pearl River Fisheries Research Institute, Chinese Academy of Fishery Sciences, Key Laboratory of Recreational Fisheries Ministry of Agriculture and Rural Areas, Guangdong Engineering Technology Research Center for Advanced Recreational Fisheries Guangzhou China; ^3^ Redpath Museum McGill University Montreal Quebec Canada; ^4^ Institute for Global Food Security, School of Biological Sciences Queen's University Belfast Belfast UK; ^5^ Ministry of Agriculture Key Laboratory of Freshwater Biodiversity Conservation, Yangtze River Fisheries Research Institute Chinese Academy of Fishery Sciences Wuhan China

**Keywords:** alien species, evolutionary experience, functional responses, herbivory rate, increasing temperature, phylogenetic relatedness

## Abstract

Evolutionary experience and the phylogenetic relationships of plants have both been proposed to influence herbivore–plant interactions and plant invasion success. However, the direction and magnitude of these effects, and how such patterns are altered with increasing temperature, are rarely studied. Through laboratory functional response experiments, we tested whether the per capita feeding efficiency of an invasive generalist herbivore, the golden apple snail, *Pomacea canaliculata*, is dependent on the biogeographic origin and phylogenetic relatedness of host plants, and how increasing temperature alters these dependencies. The feeding efficiency of the herbivore was highest on plant species with which it had no shared evolutionary history, that is, novel plants. Further, among evolutionarily familiar plants, snail feeding efficiency was higher on those species more closely related to the novel plants. However, these biogeographic dependencies became less pronounced with increasing temperature, whereas the phylogenetic dependence was unaffected. Collectively, our findings indicate that the susceptibility of plants to this invasive herbivore is mediated by both biogeographic origin and phylogenetic relatedness. We hypothesize that warming erodes the influence of evolutionary exposure, thereby altering herbivore–plant interactions and perhaps the invasion success of plants.

## 1 INTRODUCTION

Evolutionary experience is an important mediator of species interactions and thus commonly invoked to explain the success and impacts of biological invasions (Carthey & Banks, 2014; Ehrlich & Raven, 1964; Pearse & Altermatt, 2013; Saul & Jeschke, 2015; Verhoeven, Biere, Harvey, & Putten, 2009). A preference by generalist herbivores for novel plants (Agrawal & Kotanen, 2003; Fan, Yu, & Liu, 2013; Heard & Sax, 2013; Parker & Hay, 2005) could impose strong inhibitory effects on introduced species, consistent with the biotic resistance hypothesis (Levine, Adler, & Yelenik, 2004). In contrast, if generalist herbivores prefer familiar plants (Agrawal et al., 2005; Liu & Stiling, 2006; Xiong, Dan, Wang, Liu, & Wang, 2008), plant invasion may be promoted by a suppression of native competitors, consistent with the enemy release hypothesis (Colautti, Ricciardi, Grigorovich, & MacIsaac, 2004; Keane & Crawley, 2002). A meta‐analysis found that native generalist herbivores preferentially consumed exotic plants (Parker & Hay, 2005), whereas exotic herbivores preferentially consumed native plants (Parker, Burkepile, & Hay, 2006), conferring a competitive advantage to invading plants (Parker et al., 2006) through indirect facilitation (Simberloff & Von Holle, 1999). Such evolutionary mismatches render naïve plants particularly susceptible to herbivory (hereafter termed the “novel interaction hypothesis” (Buckley & Catford, 2016; Carthey & Banks, 2014; Saul & Jeschke, 2015; Verhoeven et al., 2009)). However, studies testing these patterns typically used only a dichotomic analysis (native vs. exotic species) while ignoring the specific biogeographic origins of the species involved; consequently, these studies did not test the effect of evolutionary exposure. Opportunities for such tests are provided when an exotic herbivore interacts with plants that have different biogeographic origins. If evolutionary exposure is important, the exotic herbivore should have different effects on the novel plants (native plants and exotic plants with different biogeographic origin) and on the familiar plants (exotic plants with the same biogeographic origin).

In addition to direct evolutionary exposure, herbivory damage may also be affected by plant phylogeny (Craft, Paul, & Sotka, 2013; Hill & Kotanen, 2009; Ness, Rollinson, & Whitney, 2011; Pearse & Hipp, 2009). However, the direction and magnitude of the relationship between plant phylogeny and herbivory pressure are still highly debated. One might hypothesize that exotic plants closely related to natives are more likely to be recognized and attacked by native herbivores and thus encounter resistance to invasion (Levine et al., 2004). Alternatively, exotic plants closely related to natives may be preadapted to existing conditions of herbivory. On the other hand, a generalist herbivore may choose plants based on traits such as nutrition content, regardless of the phylogenetic relatedness between exotic and native plants (Pearse & Hipp, 2009). Testing the feeding efficiencies of exotic herbivores across a phylogenetic gradient of plants may help resolve these opposing hypotheses.

Increasing temperatures could influence consumer–resource interactions with respect to coevolutionary history (Diamond & Kingsolver, 2012). For example, Diamond and Kingsolver (2012) demonstrated that herbivore populations with different evolutionary exposures to host plants varied in their responses to these plants under different temperature. Studies have also linked altered temperature to the movement (Walther et al., 2009), establishment success (Chown et al., 2012), and spread (Stachowicz, Terwin, Whitlatch, & Osman, 2002) of species beyond their natural ranges. Recently, variation in temperature has been shown to influence competition, insect–plant, and predator–prey interactions involving native and exotic species (Fey & Cottingham, 2012; Fey & Herren, 2014; Lu, Siemann, Shao, Wei, & Ding, 2013). For example, Fey and Herren (2014) found that increasing temperature disproportionately benefited an exotic species compared to a native congener under threat from a shared native predator, resulting in a temperature‐dependent enemy release. If increased temperature similarly shapes novel herbivore–plant interactions, it may mediate plant invasion and impact.

In this study, we conducted functional response (FR) experiments to examine how the biogeographic origin and phylogenetic relatedness of plants influence the feeding efficiency of an exotic herbivore under increasing temperatures. The main advantage of the FR method is its derivation of critical parameters of per capita feeding ability, including attack rate, handling time, and maximum feeding rate, rather than less informative “snapshot” measurements of feeding rates by arbitrarily setting one level of resource (Dick et al., 2014). Thus, specifically in this study, we use FRs to test (a) whether an invasive generalist herbivore prefers to feed on evolutionarily novel plants; (b) whether its feeding rate is related to the phylogenetic relatedness of the host plants; and (c) how increasing temperatures affect the effects of plant biogeography and phylogeny on herbivore feeding efficiency.

## 2 MATERIALS AND METHODS

### 2.1 Experimental organisms

The golden apple snail (*Pomacea canaliculata*; Gastropoda, Ampullariidae) is native to freshwater wetlands of South America and has been introduced widely into Asia since the 1980s (Hayes et al., 2015; Xu, Fang et al., 2016). In its introduced range, it has caused significant damage to agricultural production (Cowie, 2002), wetland plants (Fang, Wong, Lin, Lan, & Qiu, 2010), and ecosystem functioning (Carlsson, Brönmark, & Hansson, 2004) and is listed among the “Top 100” invasive species by the International Union for Conservation of Nature (Lowe, Browne, Boudjelas, & Poorter, 2000). It is an omnivorous species that feeds predominantly on aquatic and semiaquatic macrophytes (Qiu & Kwong, 2009). The snails used for this study were cultured in ponds with re‐circulating water systems (5 m × 4 m × 1.5 m) at the Pearl River Fisheries Research Institute (23°04′9.42″N; 113°12′52.68″E) in Guangzhou City, China, in 2015, and fed with Carp food pellets (Composing of crude protein, amino acid, fat, calcium, and phosphorus; Zhongshan President Enterprises Co., Ltd, Zhongshan, China).

**Figure 1:**
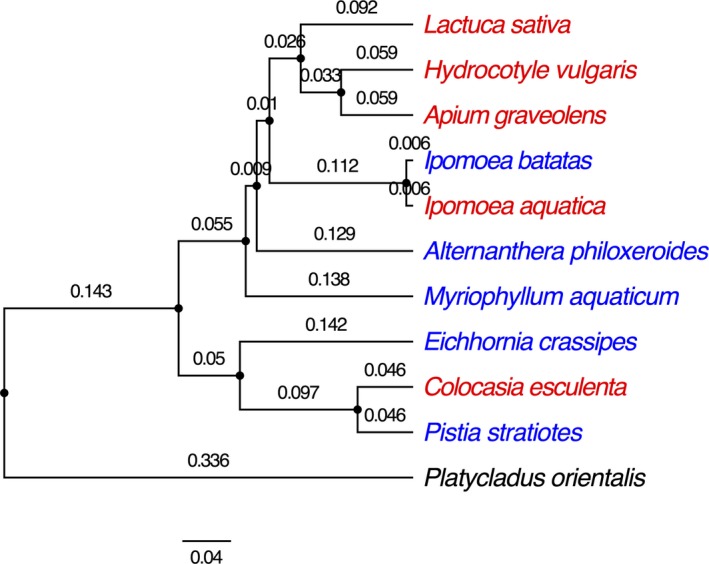
Phylogenetic relationships of the plant species used for the laboratory experiment. This molecular phylogenetic tree was constructed using Bayesian methods (see Section 2) through searching* matK* gene sequences of all these species in the GenBank. *Platycladus orientalis *was included as the out‐group species. The tiplabels of red colors were names of plant species that have different origins with the herbivorous snail while blue colors were those that have same origins with it. Branching time estimated was labeled above the branches

In the FR experiment, five plant species that share an evolutionary history with *P. canaliculata* were chosen as follows: *Alternanthera philoxeroides *(alligator weed),* Eichhornia crassipes* (water hyacinth),* Ipomoea batatas *(sweet potato),* Myriophyllum aquaticum* (parrot feather), and *Pistia stratiotes* (water lettuce). In addition, we chose the following five plant species with which *P. canaliculata* has had no evolutionary exposure: *Apium graveolens *(Chinese celery), *Colocasia esculenta *(taro), *Ipomoea aquatica* (water spinach), *Hydrocotyle vulgaris *(pennywort), and *Lactuca sativa *(lettuce) (Table 1). These plants are also phylogenetically diverse (Figure 1). Therefore, this set of host plants enables us to compare the FR of the snail on evolutionarily familiar and novel plant species and across familiar plants of different phylogenetic distance from the novel plants (Figure 1).

**Table 1:** Ten macrophytes used in the functional response experiments

Plant species	Common names	Area of origin^a^	Habitat^a^
*Alternanthera philoxeroides*	Alligator weed	South America	Aquatic
*Eichhornia crassipes*	Water hyacinth	South America	Aquatic
*Ipomoea batatas*	Sweet potato	South America	Semiaquatic
*Myriophyllum aquaticum*	Parrot feather	South America	Aquatic
*Pistia stratiotes*	Water lettuce	South America	Aquatic
*Apium graveolens*	Celery	Mediterranean	Semiaquatic
*Colocasia esculenta*	Taro	India	Semiaquatic
*Ipomoea aquatica*	Water spinach	China	Aquatic
*Hydrocotyle vulgaris*	Pennywort	Europe	Aquatic
*Lactuca sativa*	Lettuce	Mediterranean	Semiaquatic

a References: Editorial‐Board‐of‐Flora‐of‐China (1979) and Xu and Qiang (2011).

We collected all host plant species on the day of the experiment to ensure their freshness. Each of these species is common in wetlands and agricultural areas of South China, and the snail was observed to consume all these plants to some degree in the laboratory. Cultivated plants may have higher nutrient content and thus higher palatability, which could potentially confound the effect of nutritional quality with that of the evolutionary novelty; therefore, we tested differences in nutritional quality across the host plant species. We found no evidence that major nutritional (total N) and physical (dry matter content) properties of evolutionarily novel plants differed from those of evolutionarily familiar plants (see Supporting Information).

### 2.2 Functional response experiment

The FR experiment was conducted in the summer of 2015 in three consecutive experimental blocks. For each block, the experimental units were allocated to five tanks (200 cm × 70 cm × 50 cm) using a split‐plot design. The first block started at 08:00 on May 29 and ended at 20:00 on May 31 (60 hr); similarly, the second and third blocks started at 08:00 on June 4 and June 10, respectively, and ended at 20:00 on June 6 and June 12, respectively. Prior to the experiment, the snails were held without food for 24 hr to allow for standardization of hunger levels (Alexander, Dick, Weyl, Robinson, & Richardson, 2014; Xu, Mu et al., 2016). Snails with similar body size were used to minimize variation in the FR data due to body mass effects (mean body mass with shell: 13.04 ± 0.05 g). Fresh leaves of all 10 plants were picked, weighed, and allocated to experiment units. The natural air temperature range over the experimental duration was 22–26°C at nighttime and 26–32°C during daytime.

In the split‐plot design, the tank with tap water was the whole‐plot experimental unit and temperature was the whole‐plot factor. We had five whole‐plot units with water temperature controlled at 26, 28, 30, 32, and 34°C. To control the water temperature, we heated the tank using water tank heaters (mean temperatures during the experiment were 26.39 ± 0.21, 28.45 ± 0.19, 30.53 ± 0.18, 32.34 ± 0.20, and 34.38 ± 0.28°C, respectively). The tank water was maintained at 40 cm height. Within each tank, 70 boxes (12 cm × 10 cm × 6 cm) were used as subplot experimental units with plant species and plant biomass as subplot factors. These boxes consisted of 10 plant species, each having seven biomass gradients (wet weight 1, 2, 4, 6, 8, 10, and 12 g) and randomized with respect to position in tank. In each box, a golden apple snail was introduced. The experiment thus consisted of 1,050 (3 × 5 × 10 × 7) experimental units. At the end of the experiment, the leaves were removed from the boxes and allowed to dry in air for 3 hr to evaporate surface moisture before they were measured. Gross consumption by the snails was determined by subtracting final weight from initial weight. In order to characterize the potential variation of plant weights due to imbibition or air drying, we conducted a control experiment under the same experimental conditions, where the same biomass gradients of each species were included in the boxes without snails. The experiment was replicated three times with 210 (10 × 7 × 3) experimental units. After 60 hr, the leaves were removed and allowed to dry in air for 3 hr, and the natural variation at each biomass gradient for each species was subsequently determined by subtracting final weight from initial weight. The mean natural variation across seven biomass gradients for *A. philoxeroides* (alligator weed), *E. crassipes* (water hyacinth), *I. batatas *(sweet potato), *M. aquaticum *(parrot feather), *P. stratiotes *(water lettuce), *A. graveolens *(Chinese celery), *C. esculenta *(taro), *I. aquatica *(water spinach), *H. vulgaris *(pennywort), and *L. sativa *(lettuce) was −0.076 ± 0.15, 0.171 ± 0.18, 0.895 ± 0.518, 1.179 ± 0.382, 0.200 ± 0.098, 0.376 ± 0.414, 0.481 ± 0.178, 0.781 ± 0.380, 0.014 ± 0.072, and 0.571 ± 0.562 (mean ± *SD*), respectively. The net consumption by snails at each biomass gradient for each plant species was determined by subtracting the natural variation from the gross consumption. Specifically, net consumption was get using this function: [Initial weight − final weight]_treatment_ − [Initial weight − final weight]_control_. Using the measured net consumptions across each biomass gradient, we conducted the FR analyses and estimated the per capita feeding parameters.

### 2.3 Functional response analyses

Functional responses are typically employed for analyzing predator–prey dynamics (Jeschke, Kopp, & Tollrian, 2002) and have been applied less often to mechanistically understand herbivore–plant interactions (Farnsworth & Illius, 1996; Spalinger & Hobbs, 1992). In a previous study, we successfully characterized the FRs of this herbivorous snail to derive its impacts on plant resources (Xu, Mu et al., 2016). In the context of herbivores, they may rarely suffer searching limitations, owing to concentrated and ubiquitous plant resources (Spalinger & Hobbs, 1992). Thus, herbivores are unlikely to experience the classic hypothesis of competition for time between searching and handling prey (Holling, 1959), rather, cropping and chewing have been viewed as the competing processes for herbivores (Spalinger & Hobbs, 1992). Based on this assumption, and accounting for the “digestive pause” (Holling, 1966), we can easily derive Type II FRs similar to the Holling's disk function with respect to herbivores. Further, we used Rogers' random predator equation (the integral of Holling's function) to characterize FRs accounting for the nonreplacement of resource as it is consumed (Juliano, 2001):

(1) Ne=N(1-exp(a(Neh-T))).

where *Ne* is the consumed biomass of plant, *N* is the initial biomass, *a* is the “attack (cropping) rate”, *h* is the “handling (chewing and digestion) time” for per capita biomass, and *T* is the total experiment time. This recursive function can be resolved using the Lambert W function (Corless, Gonnet, Hare, Jeffrey, & Knuth, 1996):

(2) $$Ne=N-\frac{W(ahN\;\text{exp}\;(-a(T-hN)))}{\mathrm{ah}}.$$

In the laboratory, we firstly derived the FR parameters and curves for the snail toward each of the 10 plant species at each experimental temperature. The parameters *a* and *h* for the FRs were estimated using nonlinear least squares regression and the “lambert W” function of the package “emdbook” (Bolker, 2010). The estimated maximum feeding rate was calculated as 1/*h*. Then, average values of FR parameters for the five evolutionarily familiar and five novel plant species were calculated, respectively, at each temperature. To compare and visualize the FRs of the snails feeding on evolutionarily familiar and novel plant species with increasing temperature, we then constructed and compared 95% confidence intervals around the mean FR curves. Average consumption data for the snail species toward the five evolutionarily familiar and five novel plant species combined were bootstrapped, respectively, at each temperature. Then, Rogers' random predator equation was used to each data set to construct 95% confidence intervals around the mean FR curves.

### 2.4 Phylogeny reconstruction

To verify the phylogenetic structure of these species, for each of the 10 species, we searched GenBank for *matK *gene sequences, which are commonly used in published plant phylogenies (Cadotte, 2013). We included a gymnosperm *Platycladus orientalis *as the out‐group species. Sequences were aligned with ClustalW (Thompson, Gibson, & Higgins, 2003). The Bayesian phylogeny was reconstructed using BEAST version v1.8.2 (Drummond, Suchard, Xie, & Rambaut, 2012). The Bayesian MCMC chain was run for 1 million generations, and convergence was checked using Tracer version v1.6.0 (http://beast.bio.ed.ac.uk/Tracer). The maximum clade credibility tree was used to quantify phylogenetic pattern by TreeAnnotator version v1.8.2 (Drummond et al., 2012) (Figure 1).

### 2.5 Statistical analyses

Using a restricted maximum likelihood method in the “lme” function of the “nlme” package, for the laboratory experiment, we fitted linear mixed models (LMMs) to the parameters of the FRs by log transformation (Pinheiro & Bates, 2006). In these models, temperature and the origin of species (evolutionarily familiar vs. novel) were used as fixed effects. We tested their main and interaction effects on three parameters derived from the Type II FRs as follows: attack rate, handling time, and estimated maximum feeding rate. The tank was used as a random effect to describe the error structure of a split‐plot design (Crawley, 2012). The notation of the model was as follows:

$$y=\beta_{0}+\beta_{\text{temp}}x_{\text{temp}}+\beta_{\text{origin}}x_{\text{origin}}+\beta_{\text{temporigin}}x_{\text{temp}}x_{\text{origin}}+b+\varepsilon ,$$

where *y* is the parameter derived from Type II FR model (*a*, *h*, or maximum feeding rate), *β*_0_ is the intercept, *β*_temp_ and *β*_origin_ are the coefficients associated with the fixed effect variable temperature and origin. $\beta_{\text{term}\;\text{origin}}$ characterizes the interaction between these two factors. *b* is random effect (tank) depicting the error structure of the split‐plot design and *ε* is the remaining variance.

Then, in order to account for the possible effects of phylogenetic nonindependence among species on our analyses, we also conducted a PGLS (phylogenetic generalized least square regression) to examine the effect of origin and its interactions with temperature. As the PGLS required the data point must be equal to the number of tips in the phylogenetic tree (i.e., only one data point can be included for each species), we first performed the PGLS at each of the five temperature levels. Then, through averaging the data for a given species derived from different temperatures, we conducted another PGLS to test the mean effect of origin. These analyses were complished using the “gls” function in “nlme” package by hypothesizing a Brownian Motion model (Swenson, 2014).

Finally, based on the reconstructed phylogenetic tree, we calculated the mean phylogenetic distance of each familiar species to five novel species using the “cophenetic.phylo” function of the “ape” package. For example, the phylogenetic distance between the familiar species *I. batatas* and five novel species (*A. graveolens*, *C. esculenta*, *I. aquatica*, *H. vulgaris*, and *L. sativa*) was 0.236, 0.386, 0.012, 0.236, and 0.236, respectively, and then, we can obtain the mean phylogenetic distance 0.22. Using these phylogenetic distances, we fitted another LMMs to the FR parameters by log transformation. In these models, temperature and mean phylogenetic distance were fixed effects and tank was a random effect. Through these LMMs, we directly tested whether plant species closely related to novel species suffered more damage and how increasing temperature mediated the phylogenetic dependence.

All statistical analyses were performed in R, version 3.2.2 (R Core Team, 2018).

## 3 RESULTS

**Figure 2:**
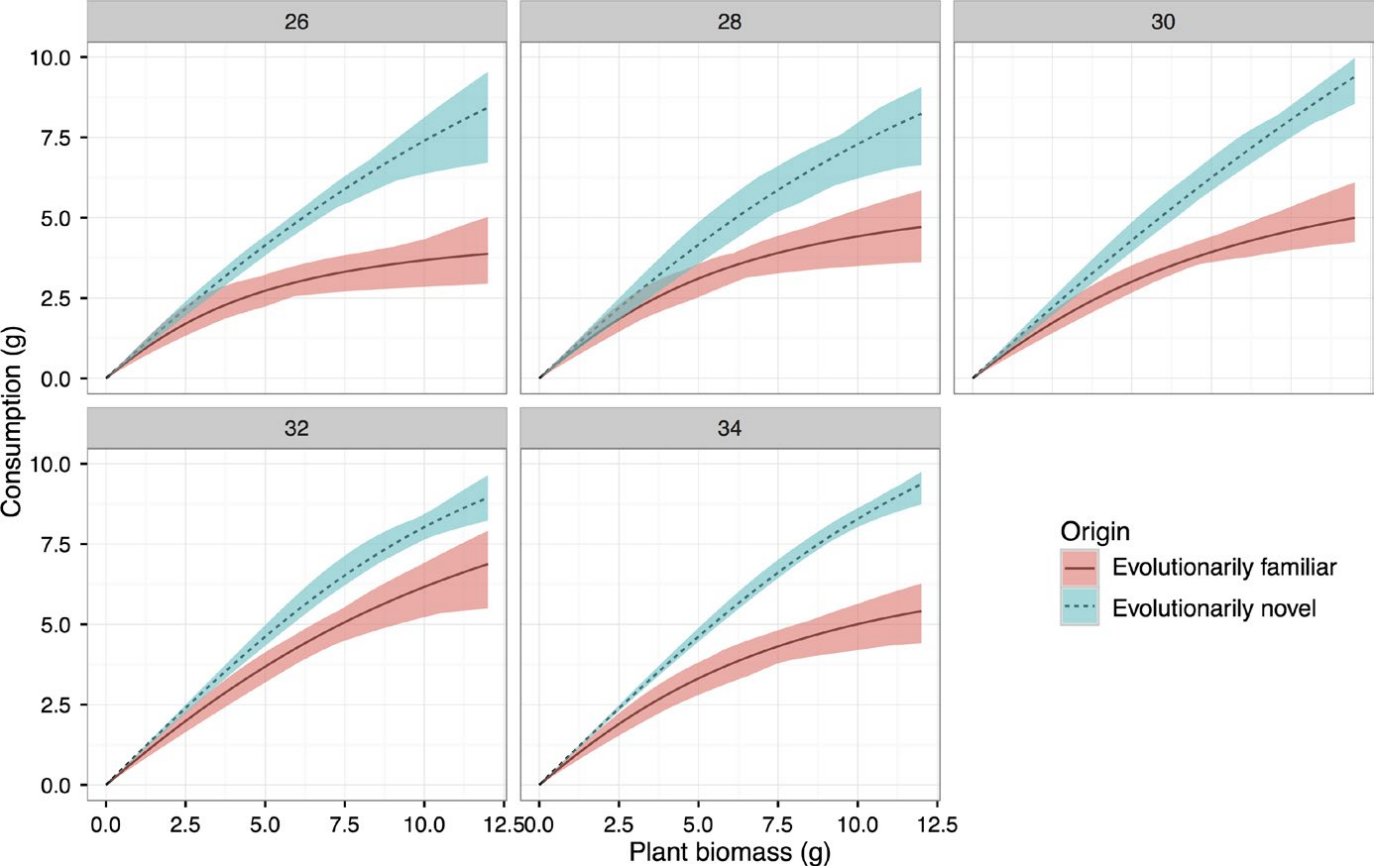
The Type II functional responses of the invasive herbivore *Pomacea canaliculata* toward five evolutionarily familiar (solid curves denotes the mean values of them) and five evolutionarily novel plant species (dashed curves denote the mean values of them) at the experimental conditions of 26, 28, 30, 32, and 34°C. The five evolutionarily familiar plants are *Alternanthera philoxeroides*, *Eichhornia crassipes*, *Ipomoea batatas*, *Myriophyllum aquaticum*, and *Pistia stratiotes*, and the five evolutionarily novel plants are *Apium graveolens*, *Colocasia esculenta*, *Ipomoea aquatica*, *Hydrocotyle vulgaris*, and *Lactuca sativa*. Shaded areas are 95% bootstrapped CIs

**Figure 3:**
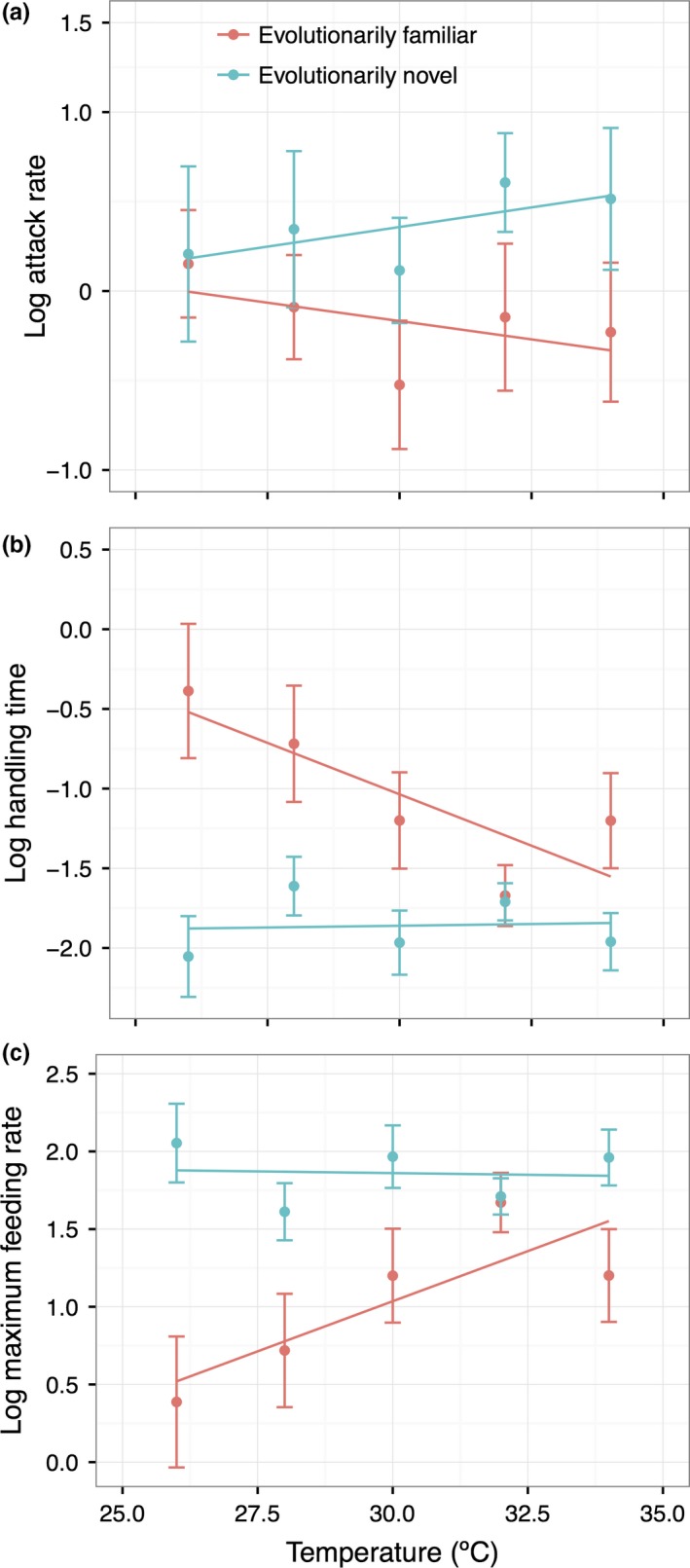
The relationships between temperature and attack rate (a), handling time (b), and maximum feeding rate (c) of the invasive herbivore *Pomacea canaliculata* toward evolutionarily familiar and novel plant species. Points represent the mean values of evolutionarily familiar or novel plant species after log transformation (*n* = 5). The linear fits come from two‐variable (temperature and origin) mixed models. Error bars represent standard errors

*Pomacea canaliculata* had markedly different FRs when consuming the evolutionarily familiar plant species than when consuming the evolutionarily novel plants (Figure 2, Supporting Information Figure S1; lack of 95% CI overlap at all temperatures), with significantly lower attack rates (Table 2, Figure 3a), higher handling times, and lower maximum feeding rates (Table 2, Figure 3b,c). There was a significant interaction effect of temperature and plant origin for the handling time and maximum feeding rate (Table 2), with higher temperature enhancing the maximum feeding rate of the herbivore when consuming the evolutionarily familiar plants, but not when consuming the evolutionarily novel plants (Table 3, Figure 3b,c). This interaction effect and the main effect of temperature did not exist for the attack rate (Table 2, Figure 3a), indicating that temperature alters the handling efficiency, but not the attack ability, of the herbivore on evolutionarily familiar plants.

**Table 2:** Results of linear mixed models predicting the log response of attack rate (*a*), handling time (*h*), and maximum feeding rate (max) derived from the Type II functional responses (FR) to temperature × origin and temperature × phylogenetic distance. “Origin” denotes whether plant species have the same biogeographic origin (five familiar species) or different biogeographic origin (five novel species) with respect to the herbivore. “Phylo” denotes the mean phylogenetic distance of each familiar species to five novel species. For FR~temp × origin model, there were 50 samples (FR parameters of 10 species in each tank × 5 tanks). For FR~temp × phylo, there were 25 samples (FR parameters of 5 familiar species in each tank × 5 tanks). In both models, there are five groups (5 tanks and temperature treatment was assigned at each tank), and thus, the tank was used as random effect to account for the autocorrelation within a tank for our split‐plot design

Models and variables	*df*_treatment_	*df*_residual_	Attack rate (*a*)		Handling time (*h*)		Maximum feeding rate (max)	
			*F*	*P*	*F*	*P*	*F*	*P*
Temp × Origin								
Temp	1	3	0.001	0.974	4.326	0.129	4.326	0.129
Origin	1	43	5.517	**0.024**	23.679	<**0.001**	23.679	<**0.001**
Temp:Origin	1	43	1.151	0.289	4.952	**0.031**	4.952	**0.031**
Temp × Phylo								
Temp	1	3	0.563	0.508	6.375	0.086	6.375	0.086
Phylo	1	18	1.021	0.326	20.731	<**0.001**	20.731	<**0.001**
Temp:Phylo	1	18	0.286	0.599	0.245	0.627	0.245	0.627

**Table 3:** Estimated effects of models predicting the log response of attack rate (*a*), handling time (*h*), and maximum feeding rate (max) derived from the Type II functional responses to plant origins × temperature and phylogenetic distance × temperature. “Origin” denotes whether plant species have same biogeographic origin (five familiar species) or different biogeographic origin (five novel species) with the herbivore. “Phylo” denotes the mean phylogenetic distance of each familiar species to five novel species

Models and variables	Attack rate (*a*)			Handling time (*h*)			Maximum feeding rate (max)		
	Estimate	*t*	*P*	Estimate	*t*	*P*	Estimate	*t*	*P*
Temp × Origin									
Familiar: Temp	−0.041	−0.733	0.467	−0.129	−3.044	**0.004**	0.129	3.044	**0.004**
Novel: Temp	0.044	0.784	0.438	0.004	0.103	0.919	−0.004	−0.103	0.919
Temp × Phylo									
26: Phylo	1.741	0.215	0.833	12.051	2.350	**0.033**	−12.051	−2.350	**0.033**
28: Phylo	−2.8438	−0.350	0.731	10.930	2.132	**0.050**	−10.930	−2.132	**0.050**
30: Phylo	−7.060	−0.870	0.398	10.298	2.008	0.063	−10.298	−2.008	0.063
32: Phylo	−4.684	−0.577	0.572	4.734	0.923	0.371	−4.734	−0.923	0.371
34: Phylo	−3.432	−0.423	0.678	11.357	2.215	**0.043**	−11.357	−2.215	**0.043**

**Figure 4:**
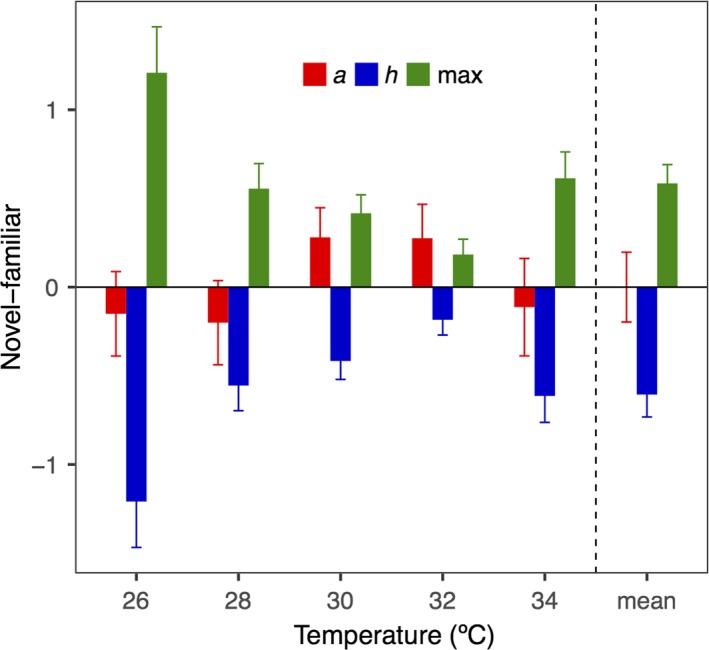
The effects of origin (evolutionary novel species‐evolutionary familiar species) on three parameters of the functional response (attack rate: *a*; handling time: *h*; and maximum feeding rate: max) at 26, 28, 30, 32, 34°C and the mean effect across temperatures in the phylogenetic generalized least square regressions (PGLS)

After accounting for the phylogeny nonindependence, the results of PGLS were consistent with that of LMMs except for the attack rate. Across all temperature, the evolutionarily familiar plants yielded higher handling time (*t = *4.804, *p* = 0.001) and lower maximum feeding rates (*t = *5.535, *p* = 0.001) than the evolutionarily novel plants, but attack rate was not significantly different between the two origins (*t = *0.001, *p* = 0.999) (Figure 4).

**Figure 5:**
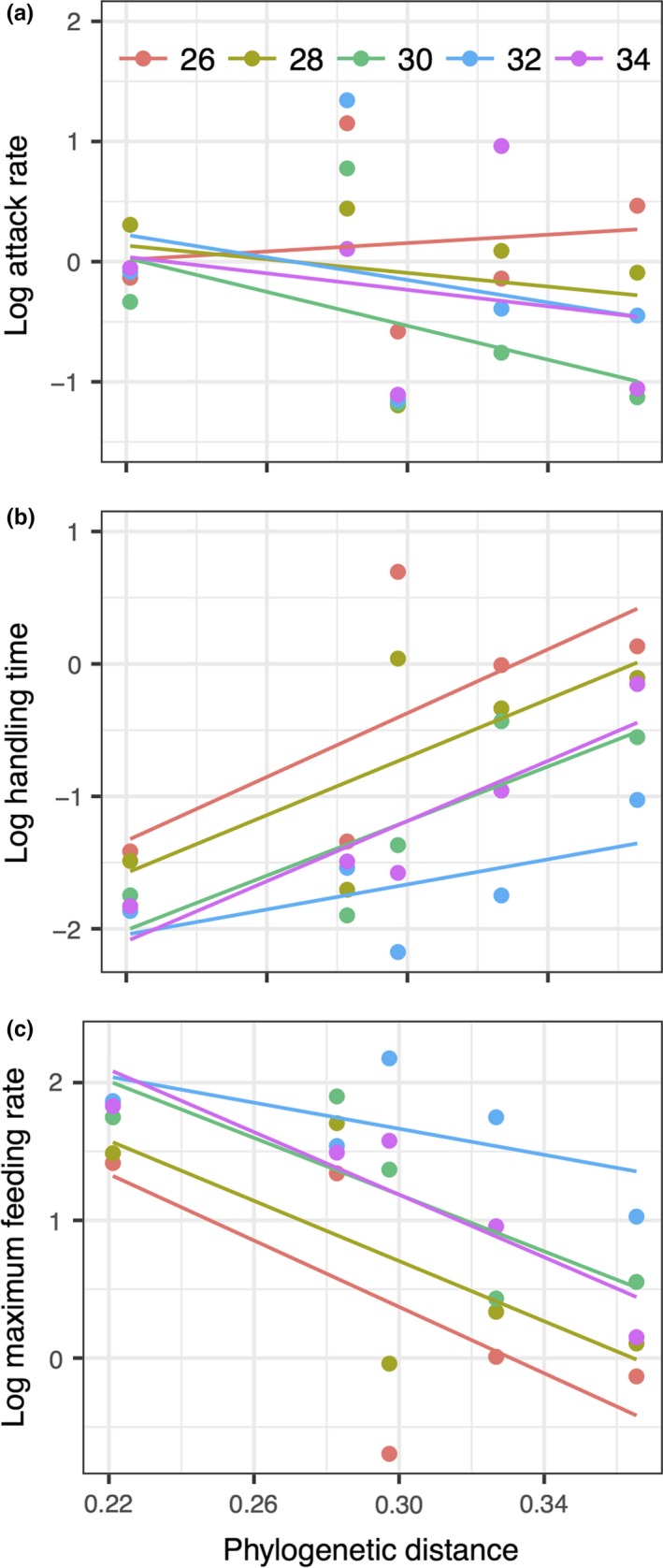
The relationships between phylogenetic distance and attack rate (a), handling time (b), and maximum feeding rate (c) of five familiar species at 26, 28, 30, 32, and 34°C. The linear fits come from two‐variable (phylogenetic distance and temperature) mixed models. Phylogenetic distance denotes the mean phylogenetic distance of each familiar species to five novel species

The mean phylogenetic distances of each familiar plant species to the five novel species significantly correlated with the handling time and maximum feeding rate of *P. canaliculata *toward these familiar species (Table 2). Plant species more closely related to novel species yielded less handling time and more herbivory pressure (Table 3, Figure 5b,c). Increasing temperature did not significantly shift this relationship (Table 2, Figure 5b,c). The phylogenetic distance, temperature, and their interaction effect did not affect the attack rate of *P. canaliculata* (Table 2, Figure 5a).

## 4 DISCUSSION

*Pomacea canaliculata* consumed evolutionarily novel plants to a greater degree than evolutionarily familiar plants, consistent with the novel interaction hypothesis (Morrison & Hay, 2011; Parker et al., 2006). Warming, however, increased the consumption rate on evolutionarily familiar plants and thus reduced the herbivory difference between evolutionarily novel and familiar plants. The feeding rate of *P. canaliculata* was higher for plant species more closely related to the palatable novel plants, and this trend was unaffected by warmer temperatures.

Our findings do not support the argument that the biogeographic origin of species has no bearing on its potential ecological impact (Davis et al., 2011; Valéry, Fritz, & Lefeuvre, 2013) and suggest that evolutionary novelty may benefit the herbivorous snail (Verhoeven et al., 2009). Five of the experimental host plants originate from South America, the same biogeographic region as *P. canaliculata*. A shared evolutionary history may have caused these plants to evolve some resistance to herbivory, whereas the plants that originated from other continents suffered higher consumption perhaps owing to lack of adaptation. Conversely, evolutionary novelty has paradoxically been cited to explain the release from natural enemies, by arguing that enemies have not been selected to counter the novel plant's defenses (Callaway & Aschehoug, 2000; Cappuccino & Arnason, 2006). Distinguishing these two contrasting processes requires consideration of underlying mechanisms such as recognition‐based and toxin‐based defenses of novel plants (Verhoeven et al., 2009). Among the plants used in our study, perhaps those familiar with *P. canaliculata* have produced phytochemicals or developed physical traits to reduce the intensity of herbivory by this snail, whereas novel plants may lack such adaptations. We focused on how increased temperature mediates this novel interaction rather than the specific mechanisms underlying it, although the latter facet deserves further exploration.

Temperature reduced the handling time and increased the maximum feeding rate of *P. canaliculata* when consuming evolutionarily familiar plants but not novel plants. This finding, on the one hand, has implications for the management of exotic species in the context of global warming. Although introduced herbivores could facilitate the success of plant invaders by suppressing their competitors in the invaded region (Parker et al., 2006), our study suggests that this facilitation might break down with increasing temperature. By contrast, Fey and Herren (2014) found that increased temperature facilitated an invasive species by promoting greater consumption of a competing native congener by a native predator. A consistent result in our experiment was the enhanced suppression by *P. canaliculata* of evolutionarily familiar plants under increasing temperature. On the other hand, such altered feeding strategies with increasing temperature likely influence the consumer fitness and long‐term population dynamics of herbivores (i.e., numerical response) (Lemoine & Burkepile, 2012; Rall, Vucic‐Pestic, Ehnes, Emmerson, & Brose, 2010). When increasing temperature enhances the metabolic rate of a herbivore but not the feeding rate, those herbivores consuming novel plants may suffering lower population growth (Rall et al., 2010). For those herbivores consuming familiar plants, increasing temperature may enhance or reduce consumer population growth, depending on variation in feeding rate and the metabolic rate. Two possible mechanisms could drive this phenomenon as follows: (a) higher temperatures may reduce the amount of toxic phytochemicals released by evolutionarily familiar plants or reduce the resistance of their tissues to predation (Stamp, Temple, Traugott, & Wilkens, 1994); (b) higher temperatures may enhance bioenergetic demands of the herbivore, causing it to increase its consumption of less palatable exotic plants. We found that handling time, rather than attack rate, on evolutionarily familiar plants varied with temperature, suggesting that temperature principally mediated the snail's efficiency of handling food rather than the attack behavior. However, an important caveat is that the attack rate was gained from the manipulated experiment with snail and plants being put in the small space. This would result in a higher value compared to that in the natural condition, in which a snail might spend more time to search for the plants. This might be a reason why we did not find the effect of temperature on this feeding parameter, given that they probably always had a “saturated” attack rate.

A significant phylogenetic dependence of diet was observed in our study. Not only did the invasive herbivore preferentially feed on evolutionarily novel native plants, it preferred the exotic plants closely related to the natives. Further, this relationship between plant phylogeny and herbivory was not influenced by the increasing temperature. When we consider novel interactions and the biogeographic origin of consumers, an intriguingly contrasting pattern may emerge in which native herbivores prefer novel plants and thus may reduce plant invasion success, whereas exotic herbivores prefer native plants and may increase plant invasion success. For example, Ricciardi and Ward (2006) found that native herbivores generally exerted stronger negative effects on the survival of exotic plants that were distantly related to native plants; whereas, in our study, an exotic herbivore had lower per capita effects on exotic plants that were distantly related to the natives. These patterns highlight the importance of considering both biogeographic context and the phylogenetic relationships of species involved in herbivore–plant interactions.

An impediment to assessing and comparing the ecological impacts of invasions is the lack of standardized methods for measuring and interpreting per capita effects (Dick et al., 2014; Ricciardi, Hoopes, Marchetti, & Lockwood, 2013). By using FR experiments, we can better depict resource use and avoid the many pitfalls of “snapshot” assessments where resource levels are arbitrarily fixed (Dick et al., 2014; Xu, Mu et al., 2016). In our study, both handling time and maximum feeding rate varied with plant origin and phylogeny, and the effect of origin was mediated by increasing temperature. However, attack rate was only affected by origin, but not by plant phylogeny and temperature. To explain this result, it would be necessary to explore the feeding behavior of the herbivore and bioenergetic tradeoffs (Jeschke et al., 2002; Spalinger & Hobbs, 1992). These distinct responses may indicate that the invertebrate herbivore has not yet evolved the ability to identify and distinguish temperature‐altered phytochemicals and therefore attacks plants without much discrimination while still exhibiting differential handling efficiencies. Alternatively, warming may enhance enzyme activity related to handling efficiency, but not attack behavior. In any case, the observed changes of handling time and maximum feeding rate indicate that plant origin and phylogenetic relationship can affect invasive herbivore–plant interactions, and such relationships are likely to be altered with increasing temperature.

## CONFLICT OF INTEREST

None declared.

## AUTHOR CONTRIBUTIONS

XDM and MX designed the experiments and wrote the first draft of the manuscript. XDM, MX, DL, and HW conducted the experiments and assembled the data. XDM, MX, AR, JTAD, YCH, and QWW performed data analysis and revised the manuscript.

## Data Availability

Data files that support this study can be obtained from: https://doi.org/10.5061/dryad.8js8vp0
