# Supplementary material for: The influence of warming on the biogeographic and phylogenetic dependence of herbivore–plant interactions
Source: Ecol Evol. 2019 Jan 28;9(4):2231–41. doi: 10.1002/ece3.4918 (PMC6392400; doi:10.1002/ece3.4918)
Supplement: Supplementary file 1 [file ECE3-9-2231-s001.docx]

**Supplementary material**

We measured nutrient (Total N, total P) and physical (DMC: dry matter content) composition data for each of our 10 experimental plants. We used these data to test for potential property differences between novel and familiar plants. DMC was determined through thermostatic drying method; total N was determined by H_2_SO4-H_2_O_2_-diffusion method; total P was determined by vanadium molybdate yellow colorimetric method. We used respectively linear regression and phylogenetic generalized least square regression (PGLS) to test the effects of N, P and DMC on the FR. The Bayesian phylogeny was reconstructed for these 10 plant species using BEAST through searching GenBank for *matK* gene sequences.

**Table S1** Nutritional and physical properties of 10 plants used in the experiment.

| Plant Species | Evolutionary property | DMC (g/kg) | N (g/kg) | P (g/kg) |
| --- | --- | --- | --- | --- |
| *Alternanthera philoxeroides* | Familiar | 830.36 | 24.04 ± 2.22 | 2.20 ± 0.03 |
| *Eichhornia crassipes* | Familiar | 883.00 | 36.35 ± 4.62 | 3.20 ± 0.08 |
| *Ipomoea batatas* | Familiar | 928.50 | 45.01 ± 5.90 | 3.36 ± 0.09 |
| *Myriophyllum aquaticum* | Familiar | 931.56 | 39.58 ± 4.44 | 4.77 ± 0.16 |
| *Pistia stratiotes* | Familiar | 920.04 | 25.53 ± 2.92 | 4.06 ± 0.19 |
| *Apium graveolens* | Novel | 929.32 | 30.23 ± 4.07 | 2.95 ± 0.15 |
| *Colocasia esculenta* | Novel | 858.25 | 45.53 ± 5.56 | 5.54 ± 0.29 |
| *Ipomoea aquatic* | Novel | 925.14 | 51.19 ± 5.34 | 5.11 ± 0.08 |
| *Hydrocotyle vulgaris* | Novel | 946.01 | 25.87 ± 3.64 | 4.31 ± 0.08 |
| *Lactuca sativa* | Novel | 930.66 | 41.77 ± 5.29 | 4.05 ± 0.23 |

**Linear regression in R:**

>anova(lm(log(N)~origin, data=nutrient))

Df Sum Sq Mean Sq F value Pr(>F)

origin 1 0.04225 0.042255 0.5322 **0.4865**

Residuals 8 0.63518 0.079397

>anova(lm(log(P)~origin, data=nutrient))

Df Sum Sq Mean Sq F value Pr(>F)

origin 1 0.13400 0.13400 1.8516 **0.2107**

Residuals 8 0.57896 0.07237

>anova(lm(log(DMC)~origin, data=nutrient))

Df Sum Sq Mean Sq F value Pr(>F)

origin 1 0.00115 0.00115 0.6034 **0.4596**

Residuals 8 0.01529 0.00191

**PGLS in R:**

**Library(nlme)**

>anova(gls(log(N)~origin, correlation=corBrownian(phy=phylo.new), data=nutrient))

Denom. DF: 8

numDF F-value p-value

(Intercept) 1 366.1879 <.0001

origin 1 4.4128 **0.0689**

>anova(gls(log(P)~origin, correlation=corBrownian(phy=phylo.new), data=nutrient))

Denom. DF: 8

numDF F-value p-value

(Intercept) 1 62.08763 <.0001

origin 1 29.69925 **6e-04**

>anova(gls(log(DMC)~origin, correlation=corBrownian(phy=phylo.new), data=nutrient)

Denom. DF: 8

numDF F-value p-value

(Intercept) 1 53439.37 <.0001

origin 1 0.19 **0.6705**

**Figure S1.** The type II functional responses of the invasive herbivore *Pomacea canaliculata* toward 5 evolutionarily familiar plant species (upper row: *Ipomoea batatas, Myriophyllum aquaticum, Alternanthera philoxeroides,* *Pistia stratiotes* and *Eichhornia crassipes*) and 5 evolutionarily novel plant species (lower row: *Apium graveolens,* *Lactuca sativa, Ipomoea aquatica,* *Hydrocotyle vulgaris* and *Colocasia esculenta,*) at the experimental conditions of 26, 28, 30, 32 and 34 ºC. The points were mean values of three repetitions, bars denote the standard errors and the solid lines were the fitted FR curves.
